# Phenolics from *Mikania micrantha* and Their Antioxidant Activity

**DOI:** 10.3390/molecules22071140

**Published:** 2017-07-08

**Authors:** Li-Mei Dong, Xu-Chao Jia, Qing-Wen Luo, Qiang Zhang, Bi Luo, Wen-Bin Liu, Xu Zhang, Qiao-Lin Xu, Jian-Wen Tan

**Affiliations:** 1 State Key Laboratory for Conservation and Utilization of Subtropical Agro-bioresources/Guangdong Key Laboratory for Innovative Development and Utilization of Forest Plant Germplasm, College of Forestry and Landscape Architecture, South China Agricultural University, Guangzhou 510642, China; donglimei1990@163.com (L.-M.D.); zhangxu919@hotmail.com (X.Z.); 2 Key Laboratory of Functional Foods, Ministry of Agriculture/Guangdong Key Laboratory of Agricultural Products Processing/Sericultural & Agri-food Research Institute, Guangdong Academy of Agricultural Sciences, Guangzhou 510610, China; jiaxuchao@126.com; 3 Guangdong Provincial Key Laboratory of Applied Botany, South China Botanical Garden, Chinese Academy of Sciences, Guangzhou 510650, China; luoyaa@foxmail.com (Q.-W.L.); zqiang55@126.com (Q.Z.); bluo023@163.com (B.L.); liuwenbin15@mails.ucas.ac.cn (W.-B.L.); 4 Guangdong Provincial Key Laboratory of Bio-control for the Forest Disease and Pest, Guangdong Academy of Forestry, Guangzhou 510520, China

**Keywords:** *Mikania micrantha*, phenolic compounds, antioxidant activity

## Abstract

A phytochemical study on the aerial parts of *Mikania micrantha* led to the isolation of two new phenolic compounds, benzyl 5-*O*-β-d-glucopyranosyl-2,5-dihydroxybenzoate (**1**) and (7*S*,8*R*)-*threo*-dihydroxydehydrodiconiferyl alcohol 9-acetate (**2**), together with twelve known compounds, benzyl 2-*O*-β-d-glucopyranosyl-2,6-dihydroxybenzoate (**3**), 4-allyl-2,6-dimethoxyphenol glucoside (**4**), (+)-isolariciresinol (**5**), icariol A_2_ (**6**), 9,10-dihydroxythymol (**7**), 8,9,10-trihydroxythymol (**8**), caffeic acid (**9**), *p*-coumaric acid (**10**), ethyl protocatechuate (**11**), procatechuic aldehyde (**12**), 4-hydroxybenzoic acid (**13**), and hydroquinone (**14**). Their structures were elucidated on the basis of extensive spectroscopic analysis. Except **8** and **9**, all the other compounds were isolated from this plant species for the first time. The antioxidant activity of those isolated compounds were evaluated using three different assays. Compounds **1**, **2**, **3**, **9**, **10**, **13**, and **14** demonstrated significant 2,2′-azinobis-(3-ethylbenzthiazoline-6-sulphonic acid) (ABTS) free radical cation scavenging activity ranging from SC_50_ 0.31 to 4.86 µM, which were more potent than l-ascorbic acid (SC_50_ = 10.48 µM). Compounds **5**, **9**, **11**, and **12** exhibited more potent 1,1-diphenyl-2-picrylhydrazyl (DPPH) radical scavenging activity (SC_50_ = 16.24–21.67 µM) than l-ascorbic acid (39.48 µM). Moreover, the ferric reducing antioxidant power (FRAP) of compounds **2**, **5**, **9**, and **11** were discovered to be also comparable to or even more potent than l-ascorbic acid.

## 1. Introduction

**Figure 1:**
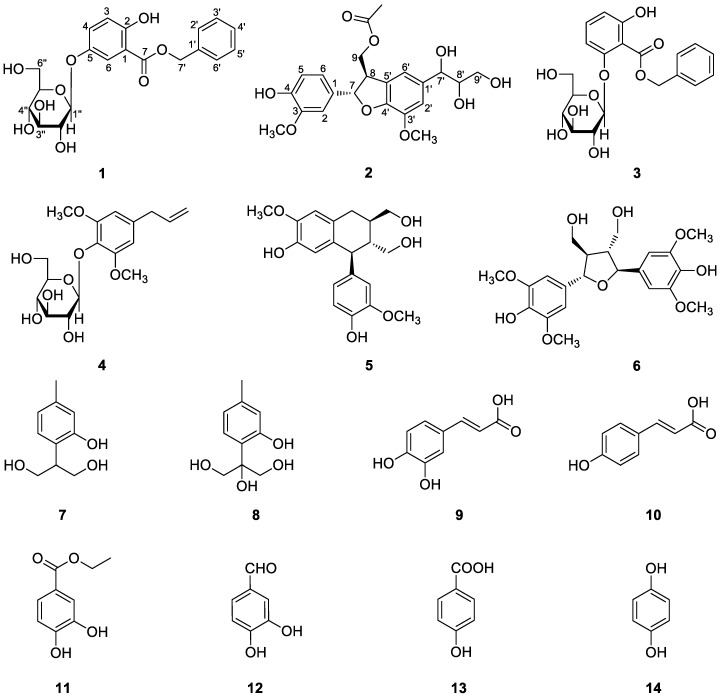
Chemical structures of compounds **1**−**14**.

*Mikania micrantha* H. B. K., belonging to the Asteraceae family, is a fast-growing perennial creeping vine indigenous to Central and South America. This plant is known as an invasive species in Southeast Asia and the Pacific region, including southern China [1]. The rapid spread of *M. micrantha* in South China has caused great damage to agriculture and forestry, as well as the ecological balance in invaded areas [1]. *M. micrantha* has long been used as a traditional herbal medicine in Jamaica to treat skin itches and athlete’s foot [2]. Previous phytochemical studies have reported some structurally diverse chemicals from this plant, including sesquiterpene lactones, flavonoids, steroids, diterpene glucosides, and phenolic compounds, some of which have shown significant bioactivities [3,4,5,6,7,8,9,10,11,12]. Our recent study on this plant also revealed a group of rare C-9 hydroxylated *ent*-kaurene diterpene glucosides [13]. During our ongoing phytochemical research on invasive plants in China [14,15,16,17,18,19], two new (**1** and **2**) and twelve known (**3**–**14**) phenolic compounds (Figure 1), were further isolated from the aerial parts of *M. micrantha*. Herein, we report the isolation and structure elucidation of these compounds, as well as their antioxidant activity.

## 2. Results and Discussion

**Figure 2:**
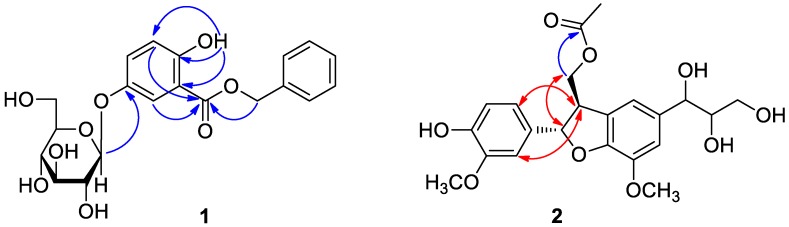
Key HMBC (blue arrows) and NOE (red arrows) correlations of **1** and **2**.

Compound **1** was obtained as a colorless syrup and deduced to have the molecular formula C_20_H_22_O_9_ from its HR-ESI-MS data, *m*/*z* 441.0971 [M + Cl]^−^ (calcd for C_20_H_22_O_9_Cl^−^, 441.0958), which required ten degrees of unsaturation. The ^1^H and ^13^C-NMR spectra of **1** (Table 1), coupled with HSQC analysis, indicated twenty carbons, including two methylenes, thirteen methines, and five quaternary carbons including one carboxyl group at δ_C_ 168.1 (C-7). The signals of δ_H_ 4.69 (1H, d, *J* = 7.6 Hz, H-1′′) and δ_C_ 60.6 (C-6′′), 69.6 (C-4′′), 73.2 (C-2′′), 77.0 (C-3′′), 76.5 (C-5′′), and 101.9 (C-1′′) indicated the presence of a β-d-glucopyranosyl moiety in the molecule. The presence of a benzyl moiety in the molecule was suggested by the proton signals at δ_H_ 7.49 (2H, m, H-2′, 6′), 7.42 (2H, m, H-3′, 5′), 7.37 (1H, m, H-4′), and 5.37 (2H, m, H-7′), and further supported by the carbon signals at δ_C_ 128.1 (C-2′, 6′), 128.6 (C-3′, 5′), 128.3 (C-4′), and 66.6 (C-7′). Besides, the presence of an ABX spin system was ascertained by the characteristic proton signals of two doublets at δ_H_ 6.94 (1H, d, *J* = 9.0 Hz, H-3) and 7.44 (1H, d, *J* = 3.1 Hz, H-6), a double doublet at δ_H_ 7.28 (1H, dd, *J* = 9.0, 3.1 Hz, H-4), and further confirmed by the ^13^C-NMR and HSQC spectra, which revealed three aromatic quaternary carbons at δ_C_ 112.9 (C-1), 155.3 (C-2), and 149.9 (C-5), and three aromatic methine carbons at δ_C_ 118.3 (C-3), 125.2 (C-4), and 117.0 (C-6). After a more carefully study of the aforementioned NMR data and taking into account the proposed molecular formula, it could be deduced that a free hydroxyl (OH) group would exist in the molecule. In the HMBC spectrum (Figure 2), correlation signals from δ_H_ 10.14 (OH-2) to δ_C_ 112.9 (C-1), 118.3 (C-3), and 155.3 (C-2) was observed, which revealed the position of the hydroxyl group at C-2. The observation of significant HMBC correlation from δ_H_ 4.69 (H-1′′) to δ_C_ 149.9 (C-5) verified the location of the glucopyranosyl moiety at C-5. The HMBC correlations from δ_H_ 6.94 (H-3) and 7.44 (H-6) to δ_C_ 168.1 (C-7) proved the connection of the carboxyl group to C-1. In addition, the ester bond linkage of C-7 with C-7′ was supported by the observation of significant HMBC correlation from δ_H_ 5.37 (H-7′) to δ_C_ 168.1 (C-7). Therefore, compound **1** was established as benzyl 5-*O*-β-d-glucopyranosyl-2,5-dihydroxybenzoate.

Compound **2**, obtained as a colorless oil, was determined to have the molecular formula C_22_H_26_O_9_ according to its HR-ESI-MS data, *m*/*z* 457.1467 [M + Na]^+^ (calcd for C_22_H_26_O_9_Na^+^, 457.1469), which required ten degrees of unsaturation. The ^1^H-NMR spectrum (Table 1) showed signals of an acetoxy methyl at δ_H_ 2.02 (3H, s), two methoxy groups at δ_H_ 3.83 (3H, s) and 3.89 (3H, s), and five aromatic protons. The ^13^C-NMR spectrum (Table 1), coupled with HSQC analysis, exhibited the signals of twenty-two carbons in total, comprising three methyls, two oxymethylenes [δ_C_ 64.3 (C-9′) and 66.7 (C-9)], nine methines (including five aromatic methanes), and eight quaternary carbons [including a carboxyl group at δ_C_ 172.7 and seven aromatic quaternary carbons]. Detailed analysis of the NMR data indicated that compound **2** closely resembled *threo*-dihydroxydehydrodiconiferyl alcohol [20,21,22], with the only difference of the hydroxyl group at C-9 being replaced by an acetoxy group in **2**. This deduction was consistent with the molecular formula and in accord with the significant HMBC correlations (Figure 2) from δ_H_ 4.33 and 4.44 (C-9) to δ_C_ 172.7. The relative configuration of H-7 and H-8 was determined to be *trans*, as supported by the NOE correlations of H-7/H-9 and H-8/H-6, H-2 (Figure 2). The absolute configuration of C-7 and C-8 in **2** was assigned to be *S* and *R* based on the positive CD Cotton effects at 292 nm and 240 nm and the negative Cotton effect at 224 nm [23,24]. Furthermore, according to literature reports about that the chemical shifts of the C-7′ and C-8′ and the value of ∆δ_C8′–C7′_ are characteristically different for the *threo* and *erythro* isomers, the relative configuration of C-7′ and C-8′ in **2** could then be determined to be *threo* based on its ^13^C-NMR data at δ_C_ 75.3 (C-7′) and 77.6 (C-8′) with the value of ∆δ_C8′–C7′_ > 2.0 ppm [25,26,27]. Hence, compound **2** was elucidated as (7*S*,8*R*)-*threo*-dihydroxydehydrodiconiferyl alcohol 9-acetate.

The twelve known compounds were identified as benzyl 2-*O*-β-d-glucopyranosyl-2,6-dihydroxybenzoate (**3**) [28], 4-allyl-2,6-dimethoxyphenol glucoside (**4**) [29], (+)-isolariciresinol (**5**) [30], icariol A_2_ (**6**) [31], 9,10-dihydroxythymol (**7**) [32], 8,9,10-trihydroxythymol (**8**) [11], caffeic acid (**9**) [33], *p*-coumaric acid (**10**) [34], ethyl protocatechuate (**11**) [35], procatechuic aldehyde (**12**) [33], 4-hydroxybenzoic acid (**13**) [36], and hydroquinone (**14**) [37] by comparison of their NMR and ESI-MS data with those reported in the literature. All the known compounds, except **8** and **9**, were isolated from this plant species for the first time.

All of the isolated compounds were measured for their antioxidant activity by using three different in vitro assays, i.e., ABTS radical cation (ABTS^•+^) scavenging assay, DPPH radical (DPPH^•^) scavenging assay, and FRAP assay, with l-ascorbic acid as a reference compound. As shown in Table 2, new compounds **1** and **2**, and known compounds **3**, **9**, **10**, **13**, and **14** demonstrated ABTS radical cation scavenging activity with SC_50_ values ranging from 0.31 to 4.86 µM, which were more potent than l-ascorbic acid (SC_50_ = 10.48 µM). Compounds **5**, **9**, **11**, and **12** exhibited more potent DPPH radical scavenging activity (SC_50_ = 16.24–21.67 µM) than l-ascorbic acid (SC_50_ = 39.48 µM). Moreover, the revealed ferric reducing antioxidant power (FRAP) of compounds **2**, **5**, **9**, and **11** were also comparable to or even more potent than the reference compound.

Generally, free radicals and reactive oxygen species (ROS) are formed unceasingly in human body and the normal presence of free radicals can produce beneficial oxidation during physiological events. However, excessive generation of free radicals in human body will bring harmful oxidation to organisms, which is recognized as a leading cause of a variety of chronic diseases such as atherosclerosis, angiocardiopathy and cancer [38,39]. It is well known that natural antioxidants can help prevent oxidation and help regulate immune function. This study, to some extent, indicate that the invasive plant *M. micrantha* is rich in structurally diverse natural antioxidants, at least in antioxidant phenolic compounds, which are potential functional chemicals beneficial for human health worthy of further investigation.

## 3. Materials and Methods 

### 3.1. General Experimental Procedures

Nuclear magnetic resonance (NMR) spectra were recorded on a Bruker DRX-500 NMR spectrometer (Bruker Biospin Gmbh, Rheistetten, Germany). Electrospray ionization mass spectrometry (ESI-MS) was measured on a MDS SCIEX API 2000 LC/MS/MS apparatus (Applied Biosystems Inc., Forster, CA, USA). High-resolution (HR) ESI-MS was measured on a Bruker Bio TOF IIIQ spectrometer (Bruker Daltonics, Billerica, MA, USA). Optical rotations were obtained on a Perkin-Elmer Model 341 polarimeter (Perkin-Elmer, Inc., Waltham, MA). UV spectra were acquired on a Perkin-Elmer Lambda 650 UV-Vis spectrometer (Perkin-Elmer, Inc., Waltham, MA, USA). Preparative HPLC was performed with an HPLC system epuipped with a Shimadzu LC-6AD pump and a Shimadzu RID-10A refractive index detector using a Shim-pack PRC-ODS C-18 column (5 µm, 20 mm × 250 mm). Medium pressure liquid chromatography (MPLC) was carried out on a CXTH P3000 instrument (Beijing Chuang Xin Tong Heng Science and Technology Co., Ltd, Beijing, China) equipped with a UV 3000 UV-Vis Detector and a C-18 column (50 µm, 50 mm × 500 mm). 

Silica gel (80–100 and 200–300 mesh, Qingdao Haiyang Chemical Co., Qingdao, China), and Sephadex LH-20 (Pharmacia Fine Chemical Co., Ltd., Oppsala, Sweden) were used for open column chromatography (CC). Thin-layer chromatography (TLC) was conducted on precoated silica gel plates (HSGF_254_, Yantai Jiangyou Silica Gel Development Co., Ltd., Yantai, China) and spot detection was performed by spraying 10% H_2_SO_4_ in ethanol, followed by heating. DPPH, ABTS and 2,4,6-Tripyridy-s-triazine (TPTZ) were purchased from Sigma-Aldrich (Shanghai) Trading Co. (Shanghai, China). Phosphate buffered saline (PBS) and l-ascorbic acid were obtained from Life technologies (Thermo Fisher Scientific, Shanghai, China) and Shanghai Boao Biotech Co. (Shanghai, China), respectively.

### 3.2. Plant Material

The aerial parts of *M. micrantha* were collected from Guangzhou, China, in July 2014, and identified by Prof. Hong-Feng Chen, South China Botanical Garden, Chinese Academy of Sciences (CAS). A voucher specimen (No. 20140705) was deposited at the Laboratory of Bioorganic Chemistry of the South China Botanical Garden, CAS.

### 3.3. Extraction and Isolation

Powdered air-dried aerial parts of *M. micrantha* (18.5 kg) were extracted three times (each time for three days) with 95% EtOH (50 L) at room temperature. The EtOH extract, after concentration in vacuo, was suspended in water (3 L) and then sequentially extracted three times each with petroleum ether (3 L) and EtOAc (3 L) to yield a petroleum ether-soluble fraction (520 g), and an EtOAc-soluble fraction (260 g) after condensation to dryness under vacuum. The EtOAc-soluble fraction was subjected to silica gel column chromatography, eluted with an increasing polarity of CHCl_3_/MeOH (from 98:2 to 70:30, *v*/*v*, each 18 L) to afford fractions E_1_–E_6_ after pooling according to their TLC profiles. Fraction E_3_ (20 g), obtained on elution with CHCl_3_/MeOH (95:5), was separated by MPLC using a decreasing polarity of MeOH/H_2_O (35:65–100:0, *v*/*v*, each 1 L) system at the flow rate of 8 mL/min to give fractions E_3-1_–E_3-20_. Fraction E_3-2_, obtained from the elution with MeOH/H_2_O (35:65), was applied on Sephadex LH-20 column chromatography with the elution of CHCl_3_/MeOH (1:4, *v*/*v*), to provide fractions E_3-2-1_–E_3-2-4_. E_3-2-4_ was purified by preparative HPLC with a Shim-pack PRC-ODS C-18 column (5 µm, 20 mm × 250 mm) using 10% acetonitrile in water (*v*/*v*) as a mobile phase at the flow rate of 8 mL/min to obtain **12** (9 mg, t_R_ = 78 min) and **6** (3 mg, t_R_ = 110 min). Fraction E_3-8_ was applied on LH-20 CC with the elution of CHCl_3_/MeOH (1:4, *v*/*v*) to furnish **5** (5 mg), and **10** (9 mg). Fraction E_4_ (18.8 g), obtained on elution with CHCl_3_/MeOH (95:5), was separated by MPLC using a decreasing polarity of MeOH/H_2_O (35:65–100:0, *v*/*v*, each 1 L) system at the flow rate of 8 mL/min to give fractions E_4-1_–E_4-18_. Fraction E_4-11_, obtained from the elution with MeOH/H_2_O (45:55), was applied on Sephadex LH-20 column chromatography with the elution of CHCl_3_/MeOH (1:4, *v*/*v*), to provide fractions E_4-11-1_–E_4-11-6_. The fraction E_4-11-3_ was further purified by preparative HPLC with a Shim-pack PRC-ODS C-18 column (5 µm, 20 mm × 250 mm) using 16% acetonitrile in water (*v*/*v*) as a mobile phase at the flow rate of 8 mL/min to obtain **2** (2 mg, t_R_ = 78 min). Fraction E_4-6_ was applied on LH-20 CC with the elution of CHCl_3_/MeOH (1:4, *v*/*v*) to furnish **7** (4 mg), **8** (18 mg), and **13** (27 mg). Fraction E_4-13_ was applied on LH-20 CC with the elution of CHCl_3_/MeOH (1:4, *v*/*v*) and then purified by HPLC using 16% acetonitrile in water (*v*/*v*) as mobile phase at 8 mL/min to furnish **4** (10 mg, t_R_ = 90 min) and **3** (13 mg, t_R_ = 100 min). Fraction E_5_ (18.6 g), obtained on elution with CHCl_3_/MeOH (90:10), was separated by MPLC using a decreasing polarity of MeOH/H_2_O (10:90–100:0, *v*/*v* , each 1 L) system at the flow rate of 8 mL/min to give fractions E_5-1_–E_5-30_. Fraction E_5-19_, obtained from the elution with MeOH/H_2_O (65:35), was applied on Sephadex LH-20 column chromatography with the elution of CHCl_3_/MeOH (1:4, *v*/*v*), to provide fractions E_5-19-1_–E_5-19-6_. The fraction E_5-19-6_ was further purified by preparative HPLC with a Shim-pack PRC-ODS C-18 column (5 µm, 20 mm × 250 mm) using 25% acetonitrile in water (*v*/*v*) as a mobile phase at the flow rate of 8 mL/min to obtain **1** (10 mg, t_R_ = 65 min). Fraction E_5-2_, was applied on LH-20 CC with the elution of CHCl_3_/MeOH (1:4, *v*/*v*) to furnish **9** (10 mg), **11** (5 mg), and **14** (3 mg).

*Benzyl 5-O-β-d-glucopyranosyl-2,5-dihydroxybenzoate* (**1**): Colorless syrup; [α${]}_{D}^{20}$ −22.50 (*c* 0.68, C_5_H_5_N); UV (C_5_H_5_N) λ_max_ nm (log *ε*) 251 (4.16), 257 (4.19); HR-ESI-MS *m*/*z* 441.0971 [M + Cl]^−^ (calcd for C_20_H_22_ClO_9_^−^, 441.0958); ^1^H-NMR (500 MHz) and ^13^C-NMR (125 MHz) data in DMSO-*d*_6_, see Table 1.

*(7S,8R)-threo-Dihydroxydehydrodiconiferyl alcohol 9-acetate* (**2**): Colorless oil; [α${]}_{D}^{20}$ + 45.71 (*c* 0.07, MeOH); UV (MeOH) λ_max_ nm (log *ε*) 282 (3.70); HR-ESI-MS *m*/*z* 457.1467 [M + Na]^+^ (calcd for C_22_H_26_NaO_9_^+^, 457.1469); ^1^H-NMR (500 MHz) and ^13^C-NMR (125 MHz) data in CD_3_OD, see Table 1.

### 3.4. Antioxidant Activity Evaluation

#### 3.4.1. ABTS Radical Cation Scavenging Assay 

The ABTS radical cation (ABTS^•+^) scavenging activity of the isolated compounds was evaluated following the procedures as previously described [40]. Briefly, potassium persulfate was added to 7 mM of ABTS^•+^, and the mixture was allowed to stand in the dark at room temperature for 12–16 h before use. The ABTS^•+^ solution was diluted with phosphate-buffered saline (PBS, pH 7.4) to provide an absorbance of 0.70 ± 0.02 at 734 nm. The diluted ABTS^•+^ solution (190 μL) was added to sample fractions (10 μL) in DMSO at different concentrations. Each treatment was conducted in triplicate. After a mixing time of 10 s and an incubation period of 6 min at 37 °C in the dark, the absorbance in each well was read at 415 nm on a Genios microplate reader (Tecan). l-Ascorbic acid was used as a positive control. The inhibitory rates of ABTS^•+^ were calculated according to the following formula: ABTS scavenging rate (%) = [1 − (absorbance of compound − absorbance of blank)/absorbance of control] × 100. SC_50_ values were calculated and expressed as means ± SD in micromolar.

#### 3.4.2. DPPH Radical Scavenging Assay

Scavenging activity of the compounds towards DPPH radicals was carried out by the method as previously described [40]. DPPH radical solution was freshly prepared with MeOH to 0.1 mM. Test compounds were dissolved in DMSO and diluted two-fold to six serial concentrations. The DPPH solution (180 μL) and sample solution (20 μL) were mixed in 96-well plates. l-Ascorbic acid was dissolved in methanol and used as a positive reference. The control group contained DMSO instead of the compound solution, and the blank group contained methanol in place of the DPPH solution. Each treatment was performed in quadruplicate. The plates were incubated at 37 °C for 30 min in the dark. The absorbance in each well was read at 515 nm on a Genios microplate reader (Tecan). The inhibitory rates of DPPH radicals were calculated according to the formula: DPPH scavenging rate (%) = [1 − (absorbance of compound − absorbance of blank)/absorbance of control] × 100. SC_50_ values (the concentrations required to scavenge 50% DPPH radicals present in the test solution) were calculated and expressed as means ± SD in micromolar. 

#### 3.4.3. FRAP Assay

Ferric reducing ability of the compounds was conducted according to the procedures as previously described [40]. FRAP reagent was made freshly by mixing 300 mM acetate buffer (pH 3.6), 10 mM TPTZ solution in 40 mM hydrochloric acid, and 20 mM aqueous ferric chloride (FeCl_3_) solution in a 10:1:1 (*v*/*v*) ratio. The TPTZ solution was prepared on the same day. Test compounds were dissolved in methanol and diluted 2-fold to six concentrations. Twenty microliters of the compound solution and 180 μL of FRAP reagent were mixed in 96-well plates. l-Ascorbic acid was dissolved in methanol and used as a positive reference. Each treatment was conducted in quadruplicate. The plates were incubated at 37 °C for 30 min in the dark. The absorbance of the product (ferrous TPTZ complex) in each well was read at 595 nm using a Genios microplate reader (Tecan Group, Mannedorf, Switzerland). One milliliter of ferrous sulfate (FeSO_4_) at six different concentrations and 1 mL of 10 mM TPTZ and 10 mL of 300 mM acetate buffer (pH 3.6) were used for a calibration curve. FRAP values were calculated and expressed as means ± the standard deviation (SD) in millimoles of Fe (II) per gram.

## 4. Conclusions

Fourteen phenolic compounds, including two new ones—**1** and **2**, were isolated from the aerial parts of *M. micrantha*. Their structures were identified by analysis of their spectroscopic data. Except **8** and **9**, all the other compounds were isolated from this plant species for the first time. Bioassays revealed that seven compounds demonstrated good ABTS radical cation scavenging activity more potent than l-ascorbic acid, and four compounds exhibited more potent DPPH radical scavenging activity than l-ascorbic acid. Moreover, the ferric-reducing antioxidant power (FRAP) of four compounds were comparable to or even more potent than L-ascorbic acid. This study indicates that the invasive plant *M. micrantha* is rich in structurally diverse phenolic compounds with functional potential beneficial for human health and is worthy of further investigation.

## Figures and Tables

**Table 1:** The ^1^H (500 MHz) and ^13^C (125 MHz) nuclear magnetic resonance (NMR) data of compounds **1** and **2**.

H/C	1 ^a^		H/C	2 ^b^	
	δ_H_ (mult, *J* in Hz)	δ_C_		δ_H_ (mult, *J* in Hz)	δ_C_
1		112.9 (C)	1		133.8 (C)
2		155.3 (C)	2	6.96 (d, 1.9)	110.6 (CH)
3	6.94 (d, 9.0)	118.3 (CH)	3		149.2 (C)
4	7.28 (dd, 9.0, 3.1)	125.2 (CH)	4		147.8 (C)
5		149.9 (C)	5	6.79 (d, 8.1)	116.2 (CH)
6	7.44 (d, 3.1)	117.0 (CH)	6	6.84 (dd, 8.1, 1.9)	120.0 (CH)
7		168.1 (C)	7	5.48 (d, 7.0)	89.7 (CH)
1′		135.6 (C)	8	3.76 (dd, 13.0, 7.0)	51.9 (CH)
2′	7.49 (m)	128.1 (CH)	9	4.33 (dd, 11.1, 7.6) 4.44 (dd, 11.1, 5.4)	66.7 (CH_2_)
3′	7.42 (m)	128.6 (CH)	1′		137.3 (C)
4′	7.37 (m)	128.3 (CH)	2′	6.98 (s)	112.9 (CH)
5′	7.42 (m)	128.6 (CH)	3′		145.4 (C)
6′	7.49 (m)	128.1 (CH)	4′		148.8 (C)
7′	5.37 (m)	66.6 (CH_2_)	5′		128.7 (C)
1′′	4.69 (d, 7.6)	101.9 (CH)	6′	6.93 (s)	116.5 (CH)
2′′	3.20 (m)	73.2 (CH)	7′	4.59 (d, 6.0)	75.3 (CH)
3′′	3.24 (m)	77.0 (CH)	8′	3.68 (dd, 4.2, 6.0)	77.6 (CH)
4′′	3.16 (m)	69.6 (CH)	9′	3.40 (dd, 11.1, 6.3) 4.44 (dd, 11.1, 4.2)	64.3 (CH_2_)
5′′	3.24 (m)	76.5 (CH)	OMe-3	3.83 (s)	56.4 (CH_3_)
6′′	3.48 (dd, 11.8, 3.5) 3.63 (dd, 11.8, 6.0)	60.6 (CH_2_)	OMe-3′	3.89 (s)	56.7 (CH_3_)
OH-2	10.14 (s)		OAc		172.7 (C)
				2.02 (s)	20.7 (CH_3_)

^a^ Recorded in DMSO-*d*_6_; ^b^ Recorded in CD_3_OD.

**Table 2:** Antioxidant activity of compounds **1**–**14**.

Compound	ABTS (SC_50_, μM)	DPPH (SC_50_, μM)	FRAP (mmol/g)
**1**	0.31 ± 0.02	>100	0.54 ± 0.03
**2**	4.19 ± 0.05	>100	12.28 ± 0.25
**3**	1.83 ± 0.07	>100	1.01 ± 0.17
**4**	>50	>100	1.41 ± 0.15
**5**	9.40 ± 0.13	21.67 ± 2.27	13.12 ± 0.23
**6**	31.81 ± 0.17	34.24 ± 0.39	5.08 ± 0.08
**7**	>50	>100	0.05 ± 0.00
**8**	6.54 ± 0.07	>100	1.13 ± 0.25
**9**	4.69 ± 0.19	16.24 ± 0.31	20.86 ± 0.24
**10**	3.48 ± 0.16	>100	3.12 ± 0.05
**11**	18.23 ± 0.36	20.57 ± 0.29	10.87 ± 0.18
**12**	9.30 ± 0.01	16.59 ± 0.24	7.79 ± 0.12
**13**	4.86 ± 0.22	>100	2.57 ± 0.07
**14**	4.57 ± 0.27	31.96 ± 1.24	8.77 ± 0.22
l-ascorbic acid	10.48 ± 0.07	39.48 ± 0.38	11.32 ± 0.13

Each value represents mean ± standard deviation (n = 3).

## Supplementary Materials

The following are available online: HR-ESI-MS and NMR spectra data of compounds **1** and **2** as supporting information.
