# Supplementary material for: Phenolics from Mikania micrantha and Their Antioxidant Activity
Source: Molecules. 2017 Jul 8;22(7):1140. doi: 10.3390/molecules22071140 (PMC6152179; doi:10.3390/molecules22071140)

Supporting Information

In this 'Supporting Information' file for the manuscript "Phenolics from *Mikania micrantha* and their antioxidant activity", MS, 1D and 2D NMR spectra of new compounds **1** and **2**, and CD spectrum of compound **2** are available here as listed below.

Contents:

Page 2–6: HR-ESI-MS, ^{1}H and ^{13}C NMR, HSQC, and HMBC spectra of **1**.

Page 7–13: HR-ESI-MS, ^{1}H and ^{13}C NMR, HSQC, HMBC, NOESY, and CD spectra of **2**.

HR-ESI-MS(-) of compound **1**

Mass Spectrum SmartFormula Report

Analysis Info

Analysis Name	D:\Data\MS\data\201601\donglimei_E9-19-6-2_neg_9_01_1327.d	Acquisition Date	1/25/2016 10:50:33 PM
Method	LC_Direct Infusion_neg_100-1000mz.m	Operator	SCSIO
Sample Name	donglimei_E9-19-6-2_neg	Instrument / Ser#	maXis 29
Comment			

Acquisition Parameter

Source Type	ESI	Ion Polarity	Negative	Set Nebulizer	0.4 Bar
Focus	Active	Set Capillary	3800 V	Set Dry Heater	180 °C
Scan Begin	100 m/z	Set End Plate Offset	-500 V	Set Dry Gas	4.0 l/min
Scan End	2000 m/z	Set Collision Cell RF	550.0 Vpp	Set Divert Valve	Waste

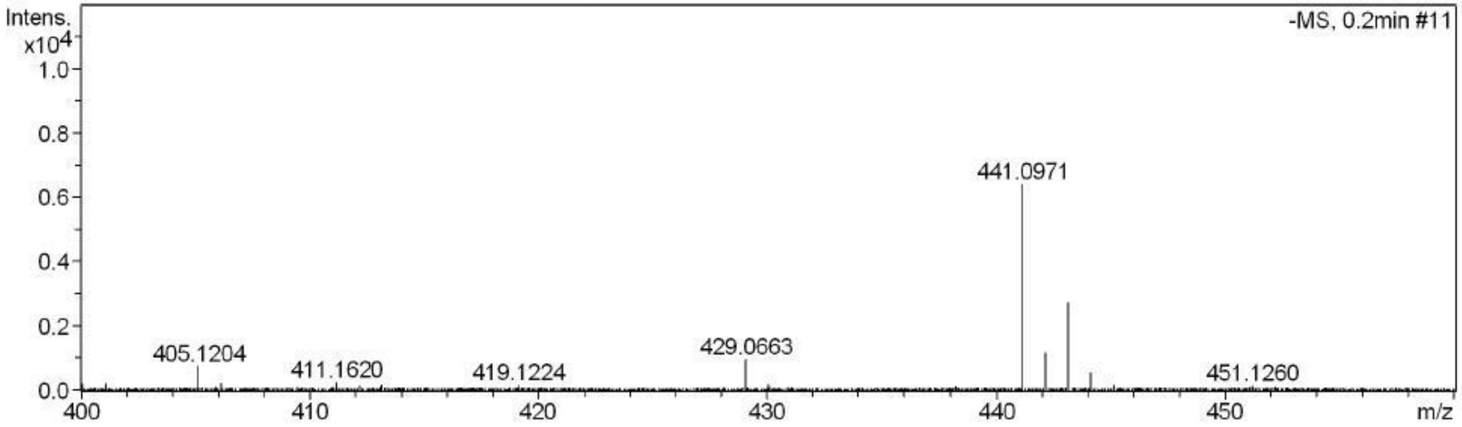

Meas. m/z	#	Formula	Score	m/z	err [ppm]	err [mDa]	mSigma	rdb	e⁻ Conf	N-Rule
405.1204	1	C 20 H 21 O 9	100.00	405.1191	-3.2	-1.3	35.0	10.5	even	ok
441.0971	1	C 20 H 22 Cl O 9	100.00	441.0958	-3.1	-1.3	33.3	9.5	even	ok
847.2237	1	C 40 H 44 Cl O 18	100.00	847.2222	-1.9	-1.6	124.6	18.5	even	ok

^{1}H NMR spectrum of compound **1**

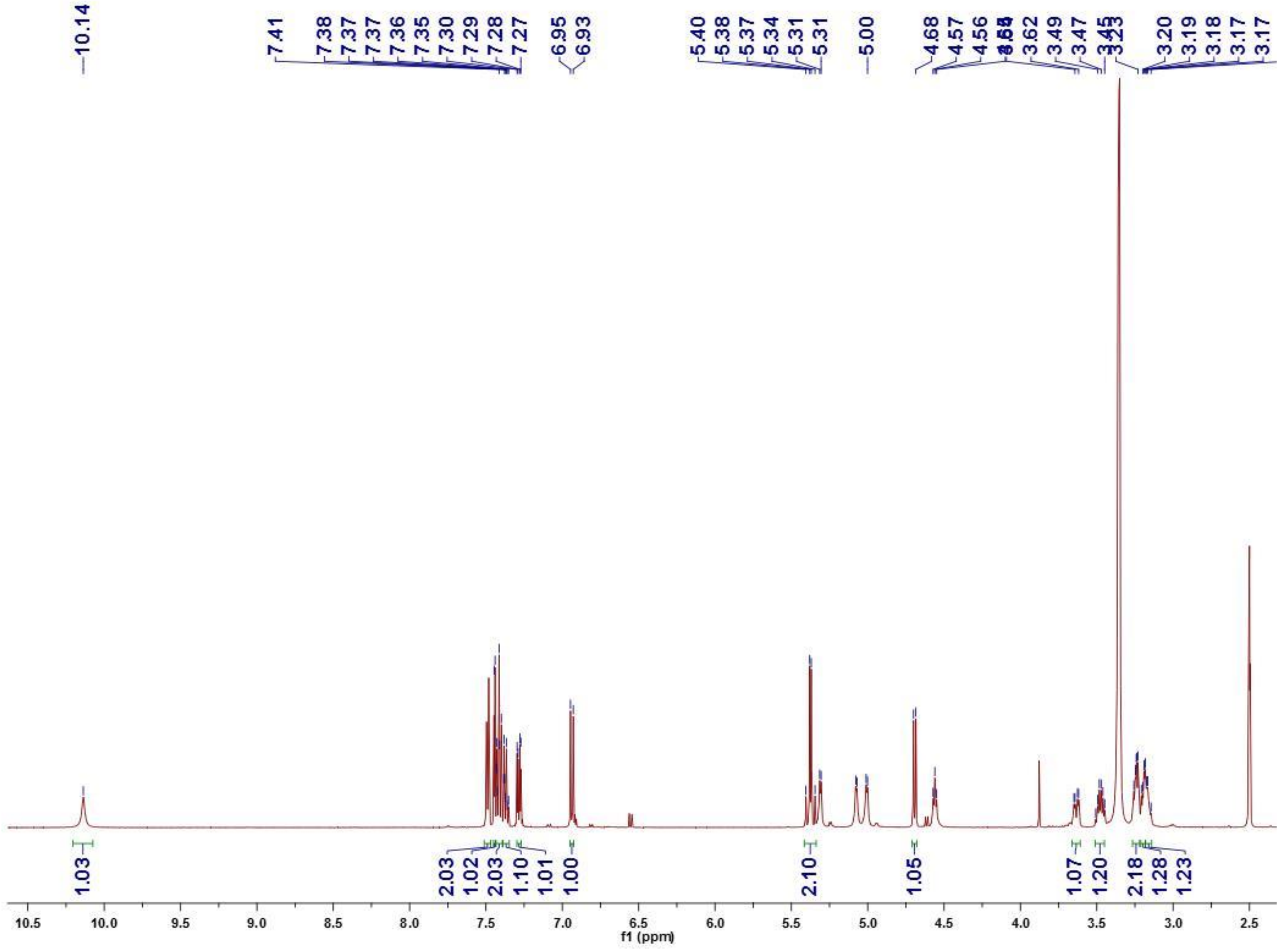

^{13}C NMR spectrum of compound **1**

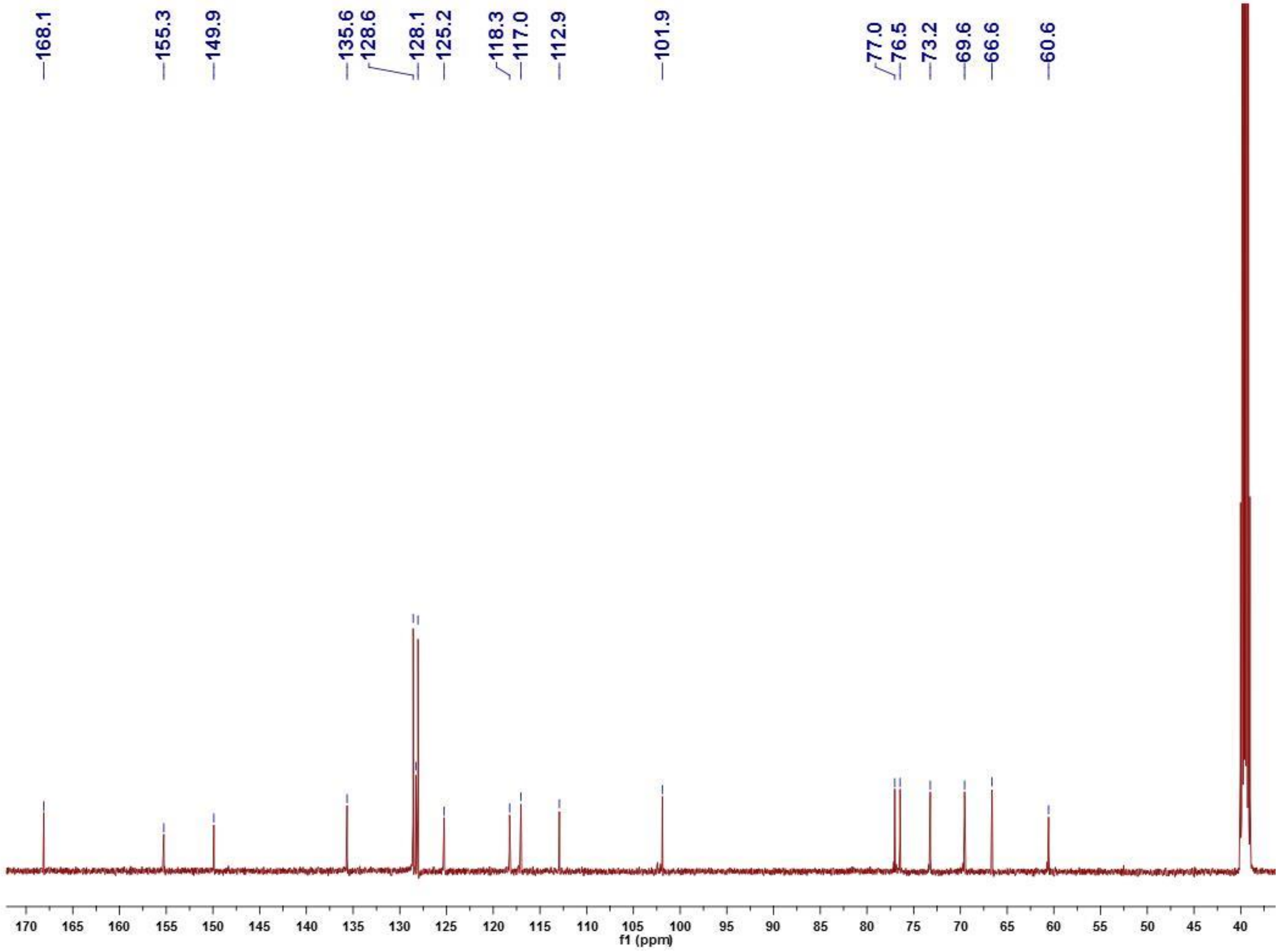

HSQC spectrum of compound **1**

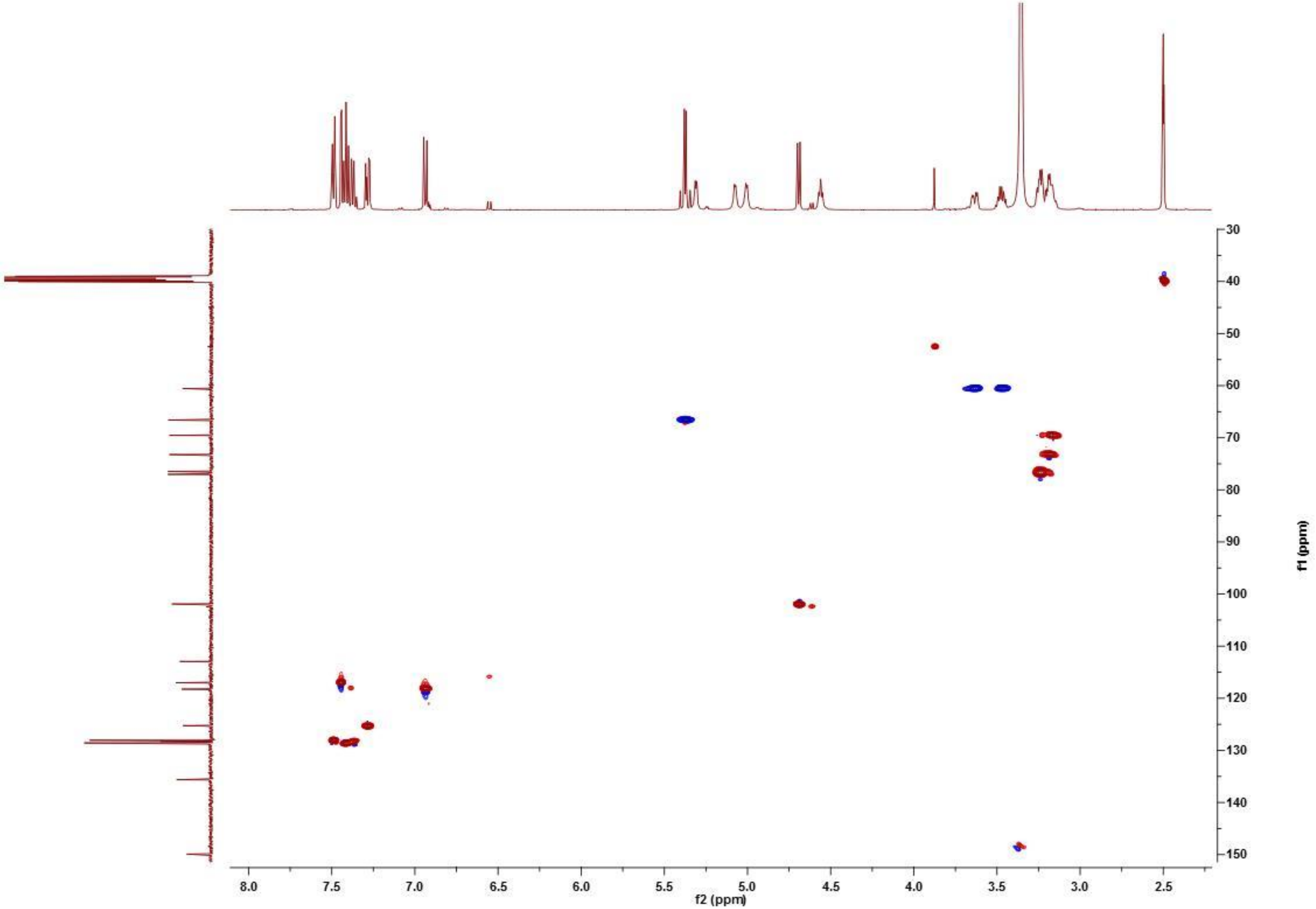

HMBC spectrum of compound **1**

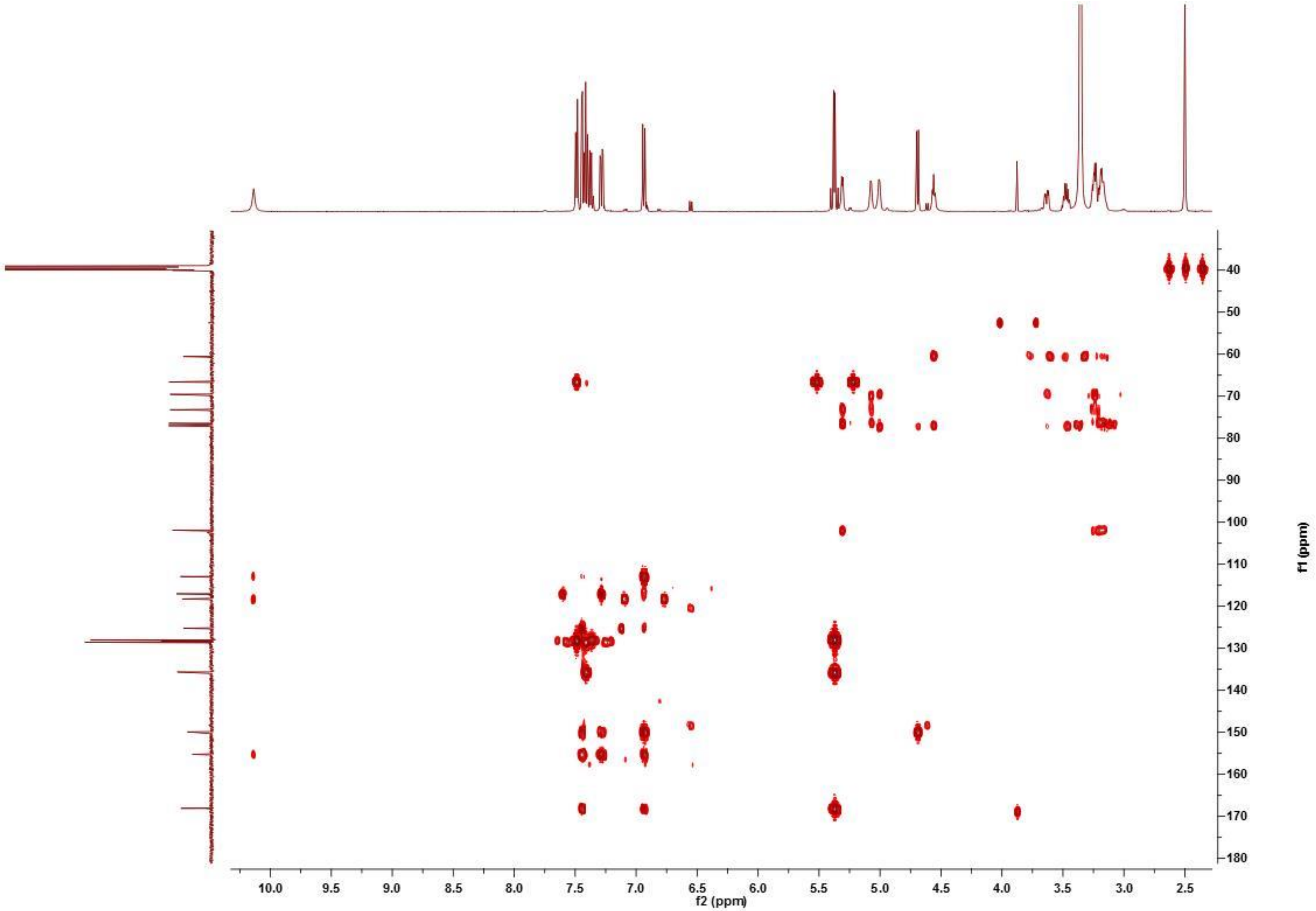

HR-ESI-MS(+) of compound **2**

Mass Spectrum SmartFormula Report

Analysis Info

Analysis Name	D:\Data\MS\data\201601\donglimei_E8-11-3-2_pos_10_01_1365.d	Acquisition Date	1/26/2016 2:32:00 AM
Method	LC_Direct Infusion_pos_100-1000mz.m	Operator	SCSIO
Sample Name	donglimei_E8-11-3-2_pos	Instrument / Ser#	maXis 29
Comment			

Acquisition Parameter

Source Type	ESI	Ion Polarity	Positive	Set Nebulizer	0.4 Bar
Focus	Active	Set Capillary	4500 V	Set Dry Heater	180 °C
Scan Begin	100 m/z	Set End Plate Offset	-500 V	Set Dry Gas	4.0 l/min
Scan End	2000 m/z	Set Collision Cell RF	800.0 Vpp	Set Divert Valve	Waste

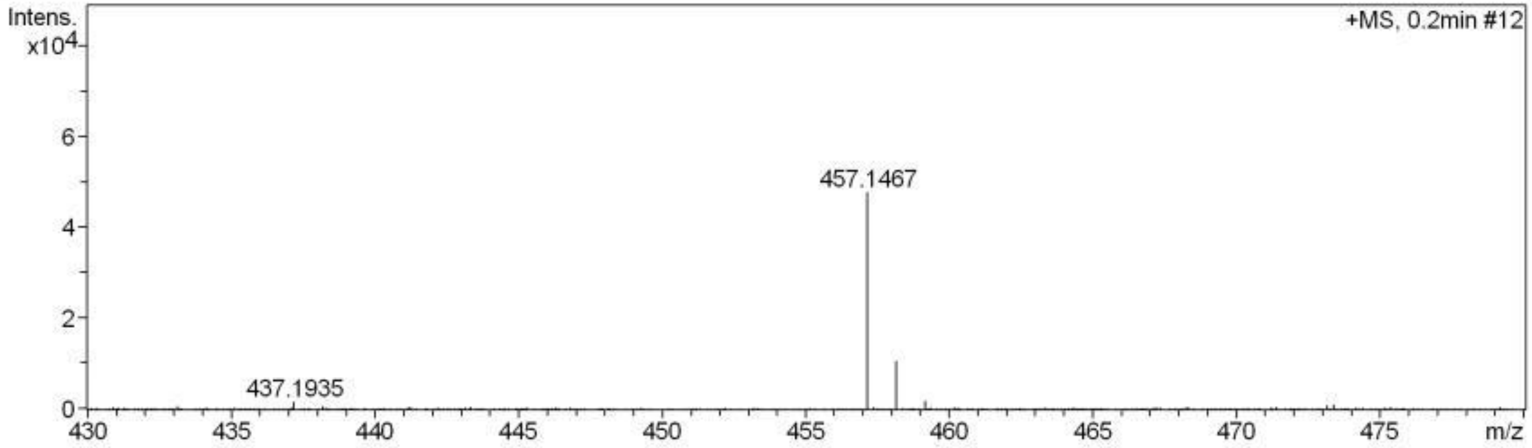

Meas. m/z	#	Formula	Score	m/z	err [ppm]	err [mDa]	mSigma	rdb	e⁻ Conf	N-Rule
457.1467	1	C 22 H 26 Na O 9	100.00	457.1469	0.4	0.2	10.1	9.5	even	ok
891.3047	1	C 44 H 52 Na O 18	100.00	891.3046	-0.1	-0.1	20.6	18.5	even	ok

^{1}H NMR spectrum of compound **2**

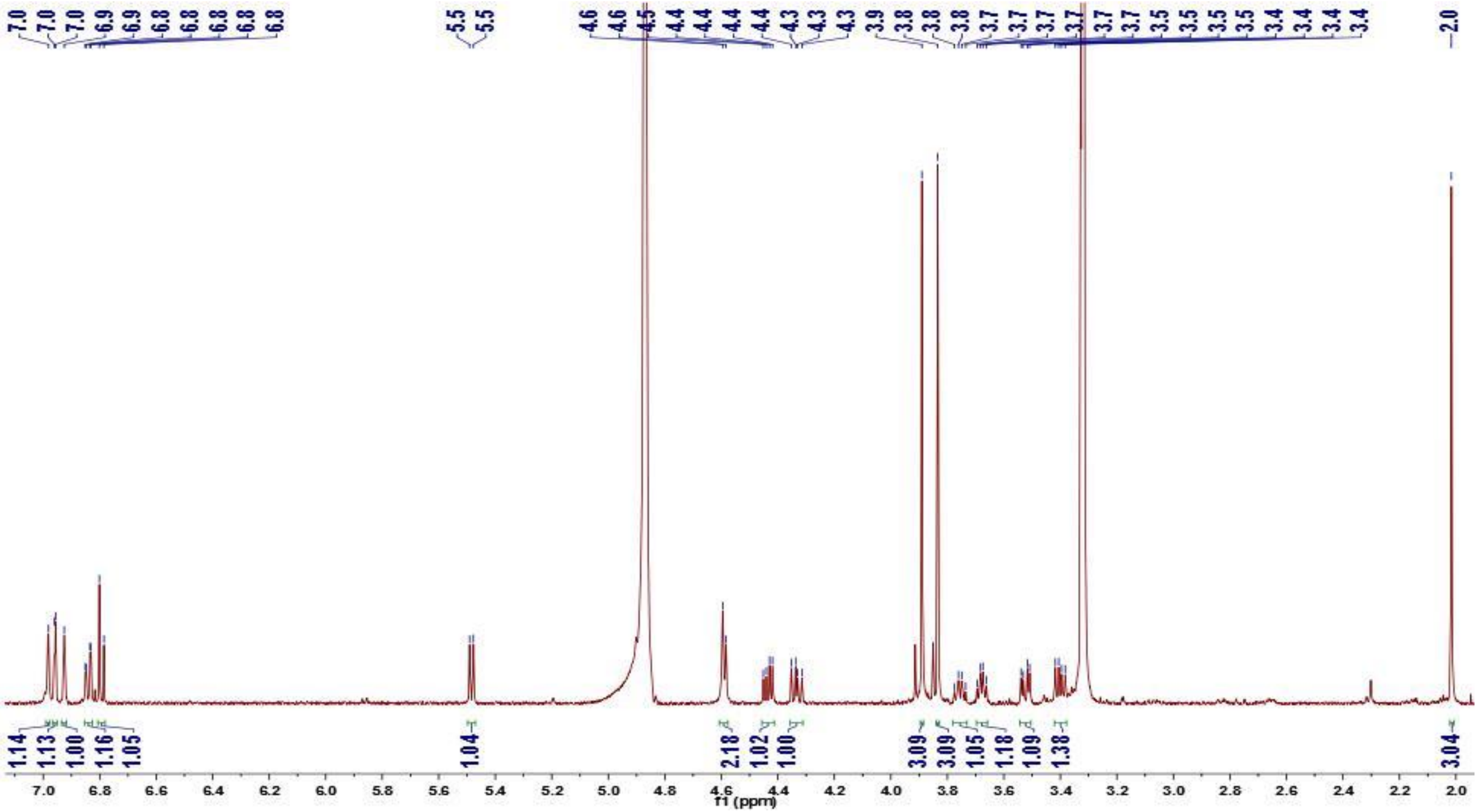

^{13}C NMR spectrum of compound **2**

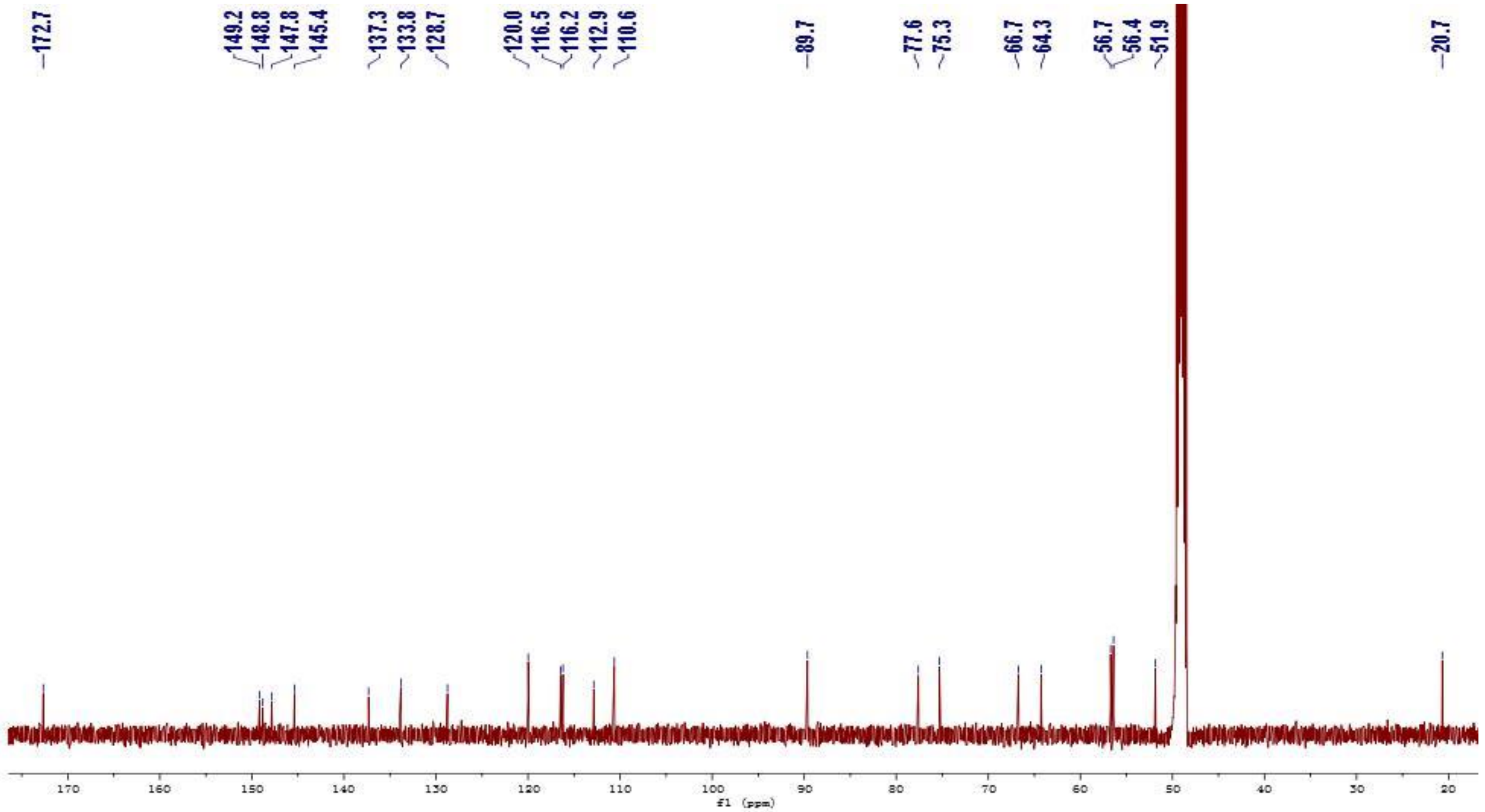

HSQC spectrum of compound **2**

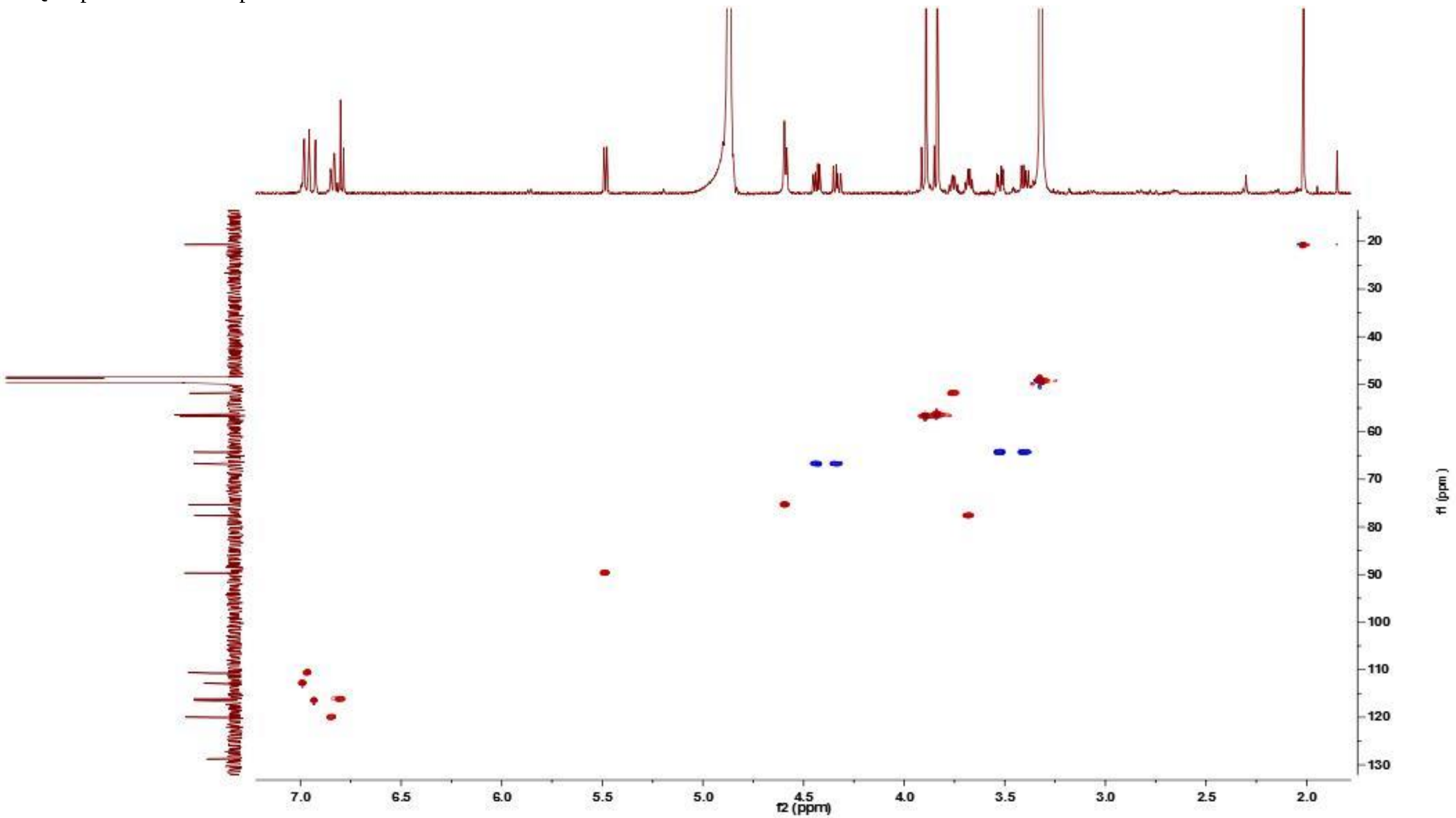

HMBC spectrum of compound **2**

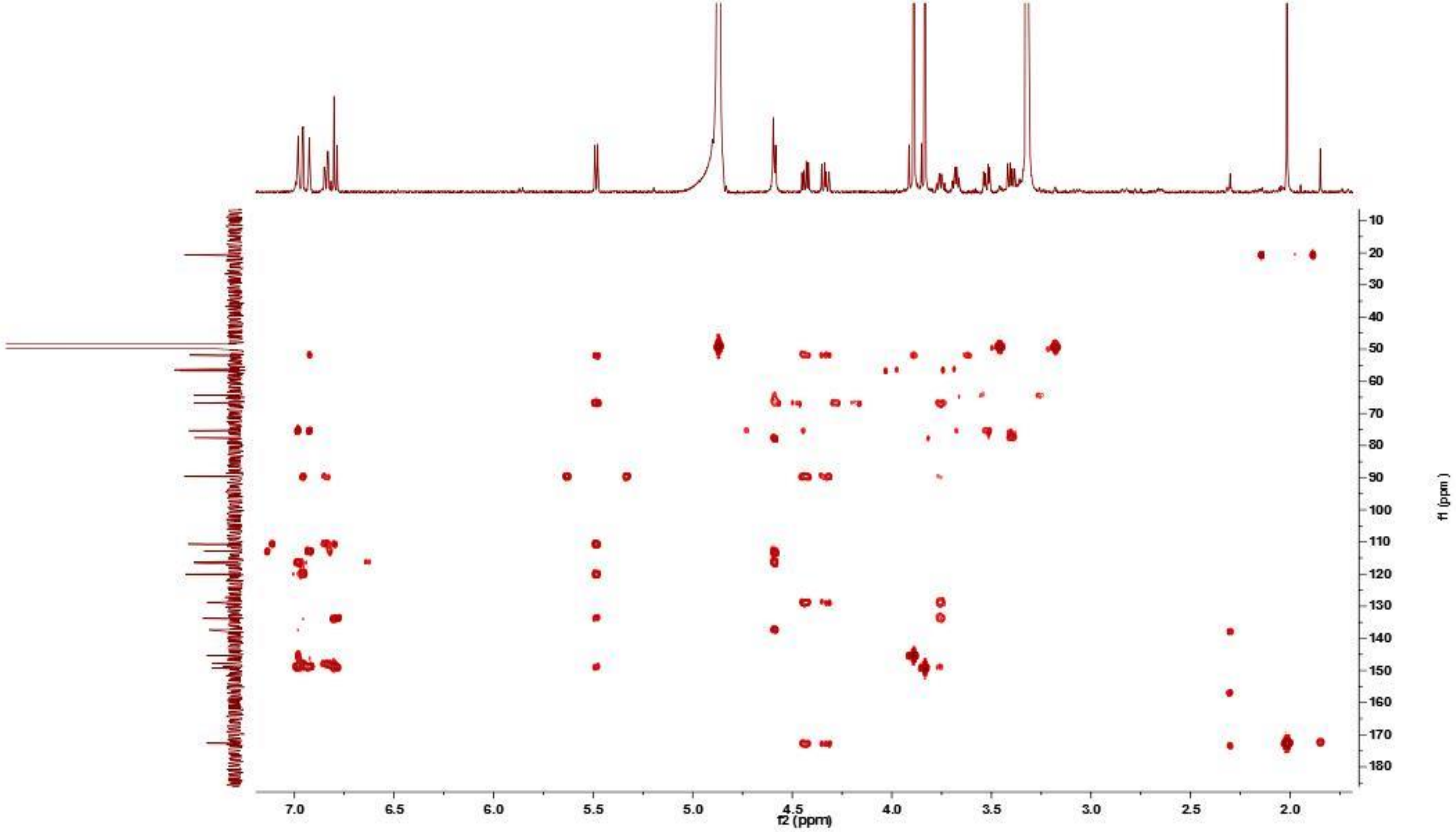

NOESY spectrum of compound **2**

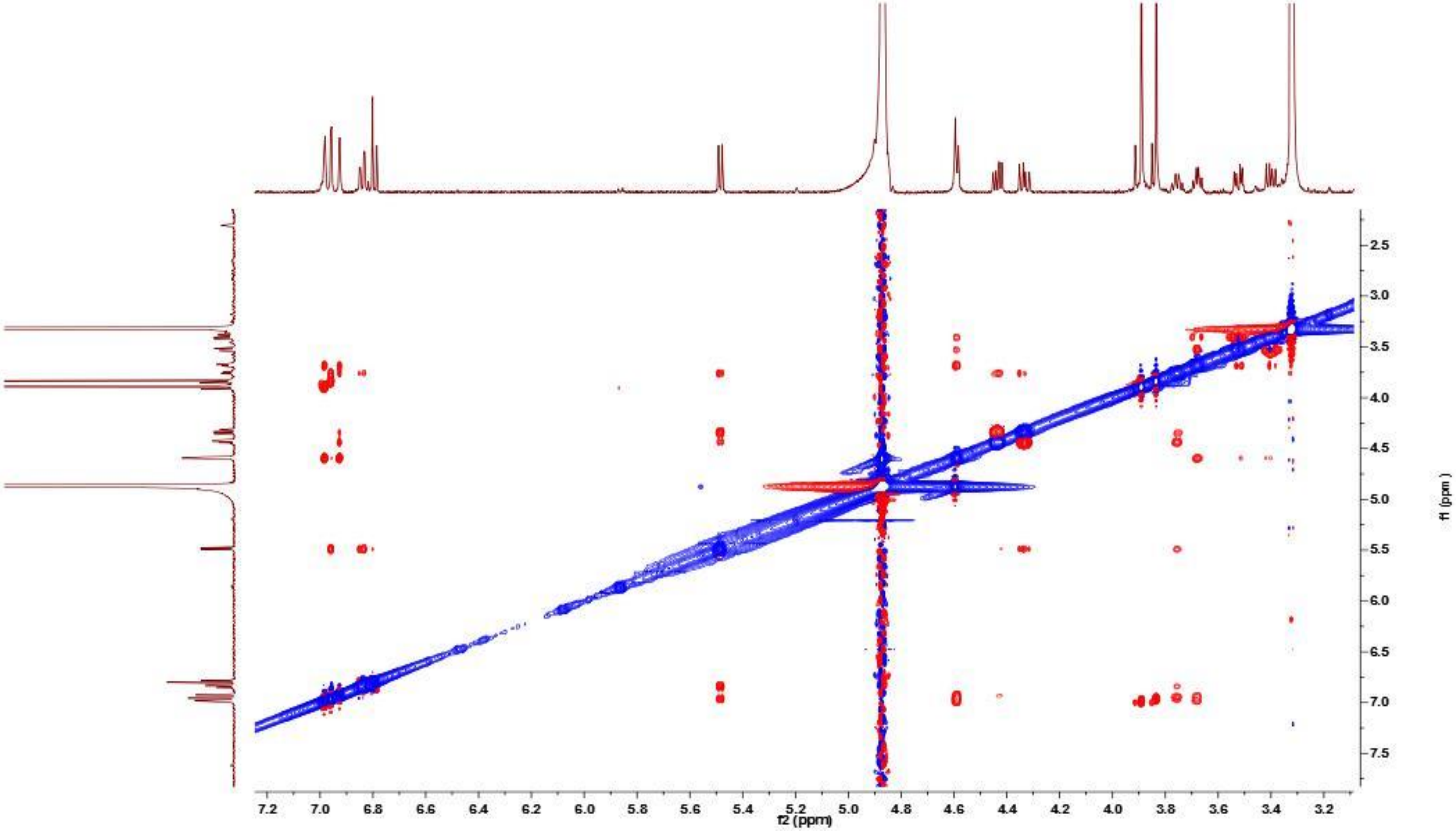

CD spectrum of compound **2**

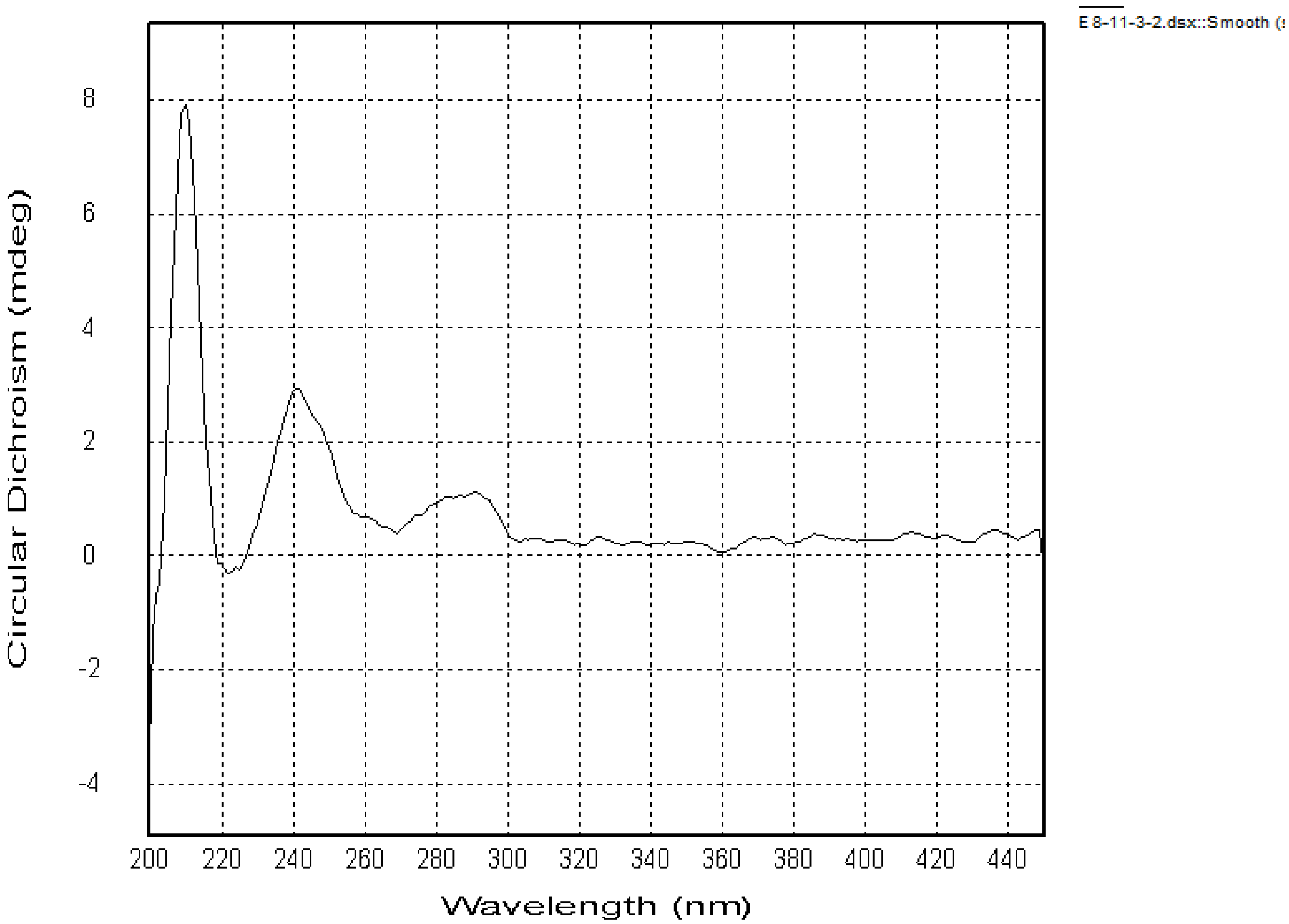

Supplement: Supplementary file 1 [file molecules-22-01140-s001.pdf]
